# Construction of molecular subgroups in childhood systemic lupus erythematosus using bioinformatics

**DOI:** 10.1097/MD.0000000000032274

**Published:** 2022-12-23

**Authors:** Jianglei Ma, Huijie Zhang, Weijiang Chu, Pengyu Wang, Huaqiu Chen, Yuanyuan Zhang, Guangming Wang

**Affiliations:** a School of Clinical Medicine, Dali University, Dali, China; b Department of Obstetrics, The Affiliated Yantai Yuhuangding Hospital of Qingdao University, Yantai, China; c Department of Endocrinology, Laizhou People’s Hospital, Yantai, China; d Department of Laboratory, Xichang People’s Hospital, Sichuan, China.

**Keywords:** bioinformatics, childhood, Gene Expression Omnibus, Gene Ontology, Kyoto Encyclopedia of Genes and Genomes, systemic lupus erythematosus

## Abstract

Systemic lupus erythematosus (SLE) is a complex autoimmune disorder. In patients with childhood SLE (cSLE), the onset of the disease occurs before 18 years of age and accounts for a high proportion of childhood autoimmune diseases. Adult SLE and cSLE differ in terms of clinical manifestations, gene expression profiles, and treatment. Because current diagnostic methods do not meet clinical requirements, researchers currently use transcriptome analysis to investigate the characteristics of the cSLE genome. In the present study, we used bioinformatics methods to genotype cSLE and identify potential therapeutic targets.

**Methods:**

The transcriptomes of 952 patients with cSLE and 94 normal controls were obtained from the Gene Expression Omnibus using unsupervised class learning to determine the genotypes in the microarray dataset, and the clinical characteristics, differentially expressed genes, and biological characteristics of the subtypes were analyzed.

**Results:**

Patients with cSLE were accordingly classified into three subgroups. Subgroup I was associated with lupus nephritis, female patients, and a high SLE disease activity index, and the disease in this subgroup was more severe than that in other subgroups. The SLE disease activity index in subgroup II was low; this subgroup may be related to lupus vasculitis. Subgroup III mostly included male patients and was associated with neuropsychiatric manifestations of lupus.

**Conclusion:**

We divided patients with cSLE into three subgroups with different characteristics based on transcriptome data. Our findings provide molecular evidence for future diagnosis and individualized treatment of cSLE.

## 1. Introduction

Systemic lupus erythematosus (SLE) is a chronic autoimmune disease characterized by the deposition of an autoimmune complex. Childhood SLE (cSLE) occurs in patients aged <18 years. Currently, the prevalence rate of cSLE is −1.89 to −34.1 per 100,000 children worldwide, with the incidence rate in females being higher than that in males (4–5:1).^[1]^ In general, cSLE is more severe than adult SLE, with a higher incidence in the kidneys, skin, and other organs.^[2]^ The quality of life of patients with SLE has improved significantly owing to the development of medical technology. Nonetheless, SLE is a complex disease, often with symptoms, autoantibodies, and susceptibility genes that overlap with those of other autoimmune diseases, resulting in its frequent misdiagnosis.^[3]^ Since the first diagnostic criteria were formulated in 1971, four different classification criteria have been developed for the diagnosis of cSLE, with the SLE classification standard developed by the Systemic Lupus Erythematosus International Collaborating Clinics in 2012 being the most commonly used classification.^[4]^ However, owing to low specificity (82.0%), this classification did not meet the requirements of the diagnostic criteria (100% sensitivity and 100% specificity), and its applicability needs to be verified in the future.^[5]^

In the previous decade, the SLE genome has been extensively studied in terms of genotyping, diagnosis, and treatment. To date, >100 SLE-susceptibility loci have been identified.^[6]^ In a multi-ethnic, large-sample study comparing patients with cSLE and lupus nephritis (LN), the authors found a statistically significant difference in human leukocyte antigen-risk loci, particularly in European patients.^[7]^ In a study on transcriptomic data obtained from patients with SLE and healthy controls, Nehar-Belaid et al^[8]^ divided patients with cSLE and SLE into four clusters based on the frequencies of the identified gene expression subsets; however, it was not specific for patients with cSLE and the precise mechanism underlying cluster identification was not mentioned. With regard to gene therapy, belimumab, a monoclonal antibody against a B-lymphocyte stimulator, has been the only FDA-approved biologic for treating SLE or cSLE for 50 years. However, the results of phase II/III trials showed that the drug is only effective for mild symptoms, as it significantly elevates anti-double-stranded DNA titers in patients and provides limited benefits in patients with refractory SLE.^[9]^ Therefore, the establishment of effective classification and targeted treatment is essential.

Using the transcriptome data of 952 patients with cSLE and 94 healthy controls from previous studies, we performed genotyping to improve cSLE diagnosis and identify possible therapeutic targets. After establishing consensus expression clusters, patients were divided into subgroups, and the differences between subgroups were compared to confirm the feasibility of cSLE genotyping. Finally, we summarized the characteristics of each subgroup to identify the possible therapeutic targets.

## 2. Materials and Methods

### 2.1. Data extraction and processing

The Gene Expression Omnibus (https://www.ncbi.nlm.nih.gov/geo/) database was searched using the keyword “systemic lupus erythematosus,” the sample cSLE dataset was retrieved, and microarray datasets (GSE27427, GSE65391, and GSE148810) and their corresponding platform files (GPL6106, GPL10558, and GPL28426, respectively) were downloaded. Each chip dataset contained information about patients with cSLE and healthy controls. R software (vX64 4.0.3; https://www.r-project.org/) was used to eliminate the batch effect of the three datasets, merge the data, and obtain the final gene expression dataset. Clinical data included age, sex, race, and SLE disease activity index (SLEDAI). All data and information in this study are from the Gene Expression Omnibus (GEO) public database. Ethical approval and informed consent of patients are not required because the data used in this study is publicly available and does not involve individual patient data or privacy.

### 2.2. Subgroup construction

The “ConsensusClusterPlus” package in R (v3.15; https://www.bioconductor.org/) was used to quantify and visualize genes and estimate the number of unsupervised classes in the dataset.^[10]^ The algorithm used was as follows: the gene expression matrix was divided up to *k* groups using the specified clustering algorithm (agglomerative hierarchical clustering, *k*-means, or a custom algorithm), and the operation was repeated for the specified number of times. The consensus value of clustering refers to the “the proportion of clustering runs in which two items are together,” and the operation results were stored in the consistency matrix of each *k* and finally processed into *k* groups using the 1 − consensus value.^[11]^ High consistency scores of each cluster in the grouping indicated that the differentially expressed genes (DEGs) in the cluster have high genetic similarity. Subsequently, gene expression subgroups were obtained and their authenticities were analyzed in detail.

### 2.3. Comparison of the clinical characteristics of subgroups

To identify differences in the clinical features of the three subgroups, we analyzed several clinical features. First, we used SPSS software (v26.0; IBM Corp., Armonk, NY) to analyze the mean ± standard deviation (SD) of patient age and SLEDAI, following which the “rstatix” package in R (v0.7.0; https://rpkgs.datanovia.com/rstatix/) was used to analyze age and SLEDAI in each subpopulation. Patient sex and race were used as categorical variables. Additionally, R software was used to calculate the proportions of the two variables in each subgroup. African American (AA), Caucasian (C), and Hispanic (H) patients were predominant among the individuals studied.

### 2.4. Screening of DEGs and protein–protein interaction (PPI) network analysis

To distinguish the specificity of each subgroup at the molecular level and investigate the possible potential therapeutic targets of each subgroup after completing the grouping, DEGs with differences >0.2 and an adjusted *P* < .05 were screened by comparing the subgroups among themselves and with the normal control group. The DEGs of each subgroup were further compared, and genes showing upregulated expression in a specific subgroup were screened (downregulated genes were excluded), yielding the specific genes of each subgroup.

To understand the relationship of each subgroup in terms of protein interactions, the highest-ranked specific genes in each subgroup were used as factors in the PPI network and transferred to the STRING website (https://stringdb.org/), with the confidence factor set to 0.4 to obtain the PPI network. Using the “Degree” algorithm in Cytoscape (v3.9.1),^[12]^ the top 10 genes with the largest number of nodes were obtained to analyze the genes in the subgroup that might have more significant biological functions.

### 2.5. Gene set enrichment analysis (GSEA) of subgroups

To determine whether the unique DEGs between different subgroups and the DEGs between subgroups and normal samples were consistent, we performed GSEA. First, Perl (v5.32.1; https://www.perl.org/) was used to convert the data in the dataset to obtain the gene list and gene set in a Perl-format file corresponding to each subgroup. These were then transferred to GSEA software (v4.1.0; https://www.gsea-msigdb.org/gsea/msigdb/) to obtain the results of the enrichment analysis of specific genes in each subgroup of the control group.

### 2.6. Construction and analysis of weighted gene co-expression network analysis (WGCNA)

WGCNA is used to analyze clusters of highly correlated genes, summarize the characteristics of closely related clusters, and correlate modules with each other and with external sample traits.^[13]^ We used the “WGCNA” package in R (v1.71; http://horvath.genetics.ucla.edu/html/CoexpressionNetwork/Rpackages/WGCNA/) to analyze the corrected gene dataset, genes specifically upregulated in each subgroup, and clinical characteristics, including age, SLEDAI, and sex. The adjacency values between all DEGs and correlation matrices were calculated using the power function, after which the topological overlap matrix (TOM) and corresponding dissimilarity (1-TOM) values were calculated.^[10]^ A gene dendrogram was generated based on the corresponding differences in the TOM, and genes with similar expression profiles were divided into co-expressed gene modules with different colors using the dynamic tree-cutting method. Finally, the correlations of gene modules with age, SLEDAI, and sex were analyzed.

### 2.7. Gene Ontology (GO) and Kyoto Encyclopedia of Genes and Genomes (KEGG) analysis

GO covers three biological aspects, namely biological process (BP), molecular function (MF), and cellular component (CC), whereas the KEGG database mainly performs enrichment analysis of molecular biological pathways. The “clusterProfiler” (v3.15; https://bioconductor.org/packages/release/bioc/html/clusterProfiler.html), “org.Hs.e.g..db” (v3.15; https://www.bioconductor.org/packages/release/data/annotation/html/org.Hs.eg.db.html), and “enrichplot” (v3.15; https://bioconductor.org/packages/release/bioc/html/enrichplot.html) packages were used for analysis, and the results are presented as bubble charts. To further understand the enrichment of DEGs in each module pathway between the control group and each subgroup, the most significantly enriched pathways in each gene module were screened from the KEGG results for analysis and comparison, and are then shown using a heat map.

### 2.8. Summarizing the characteristics of each subgroup

After the analysis of subgroups and the relevant clinical characteristics and molecular and biological levels, we summarized the results and obtained the corresponding characteristics of each subgroup.

### 2.9. Statistical analysis

Statistical analysis was performed using SPSS (v26.0; IBM Corp.), and the results are presented as the mean ± SD. R software (v4.0.3) was applied using the x86_64-pc-linux-gnu (64-bit) platform. Results were considered statistically significant at *P* < .05.

## 3. Results

### 3.1. Elimination of batch effects from transcriptome data

**Figure 1.**
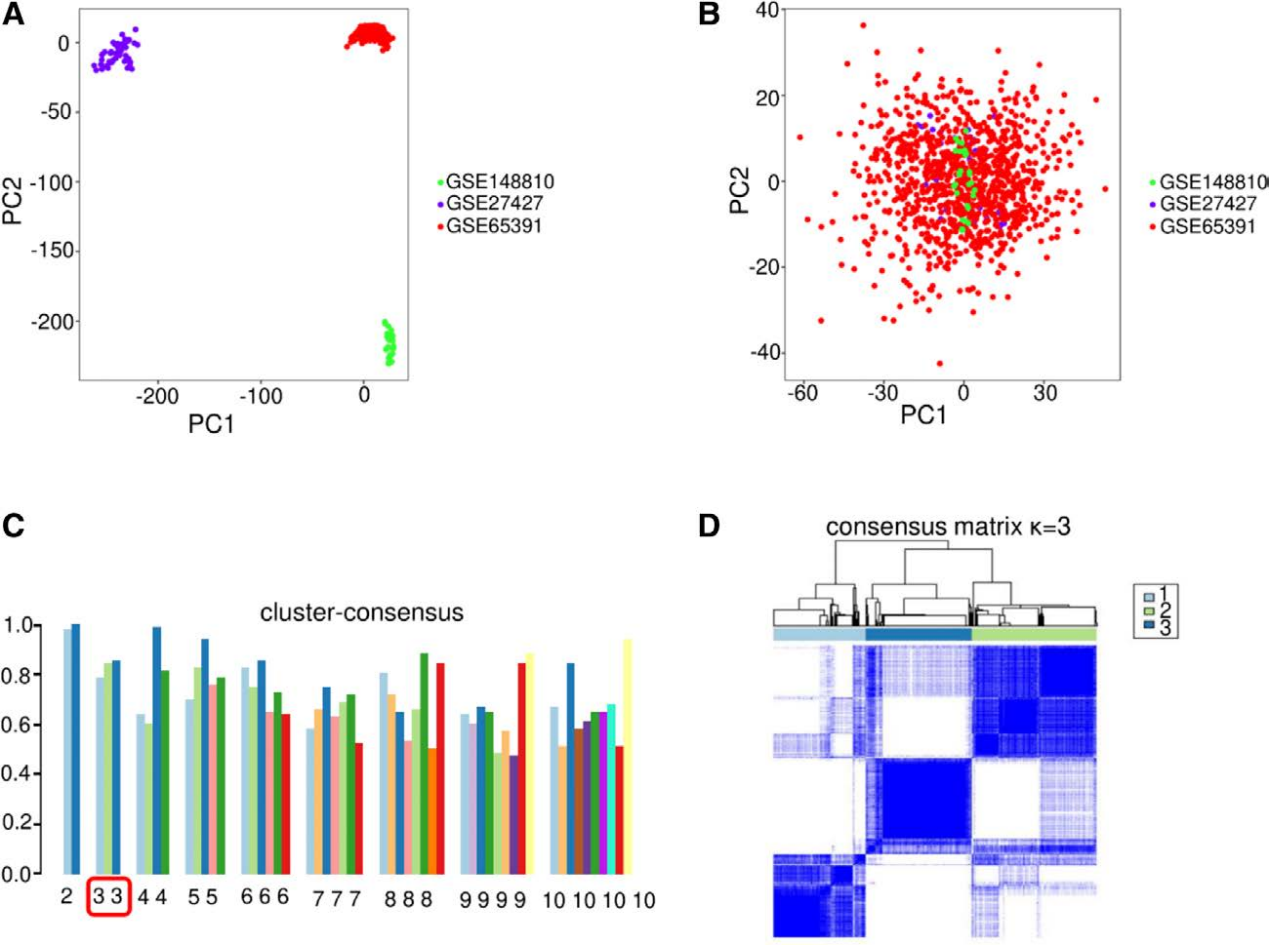
Preprocessing and clustering of three microarray datasets. (A) The three datasets showed no obvious relationship and were represented by different colors prior to eliminating the batch effect. (B) Clustering map of all genes, showing concentrated and uniform gene clusters. (C) To obtain a consistency score for the nine clusters, we used the scores for each group, which were uniform and high when divided into three clusters. The abscissa represents different clusters, and the ordinate represents the consistency score results. (D) Gene set enrichment analysis (GSEA) of the three clusters among the subgroups. A darker blue color and regular shape of the rectangle in each subgroup signify a higher correlation among differentially expressed genes (DEGs).

The unprocessed GSE 27427, GSE 65391, and GSE 148810 microarray datasets were preprocessed, and batch effects were eliminated, resulting in principal component analysis plots for all datasets (Fig. 1A and B). We observed no obvious correlation between the first three subgroups without eliminating batch effects. After processing, the sample dataset was concentrated, indicating that the entire dataset could be considered.

### 3.2. Dividing transcriptome data into three subgroups based on gene-clustering effects

According to the unsupervised learning method, the number of clusters *k* was set from two to 10, with nine clusters obtained in total. Consistency scoring on each cluster revealed that when the number of clusters was three, the scores between each group exceeded 0.75, indicating that the genes in the subgroups showed good correlation (Fig. 1C).^[10]^ The pattern map of DEG enrichment showed significant differences in the expression in each subgroup when patients with cSLE were divided into three subgroups (Fig. 1D). The number of cases in the three subgroups was as follows: subgroup I, 271; subgroup II, 367; and subgroup III, 314.

### 3.3. Analysis of the clinical characteristics between subgroups

**Figure 2.**
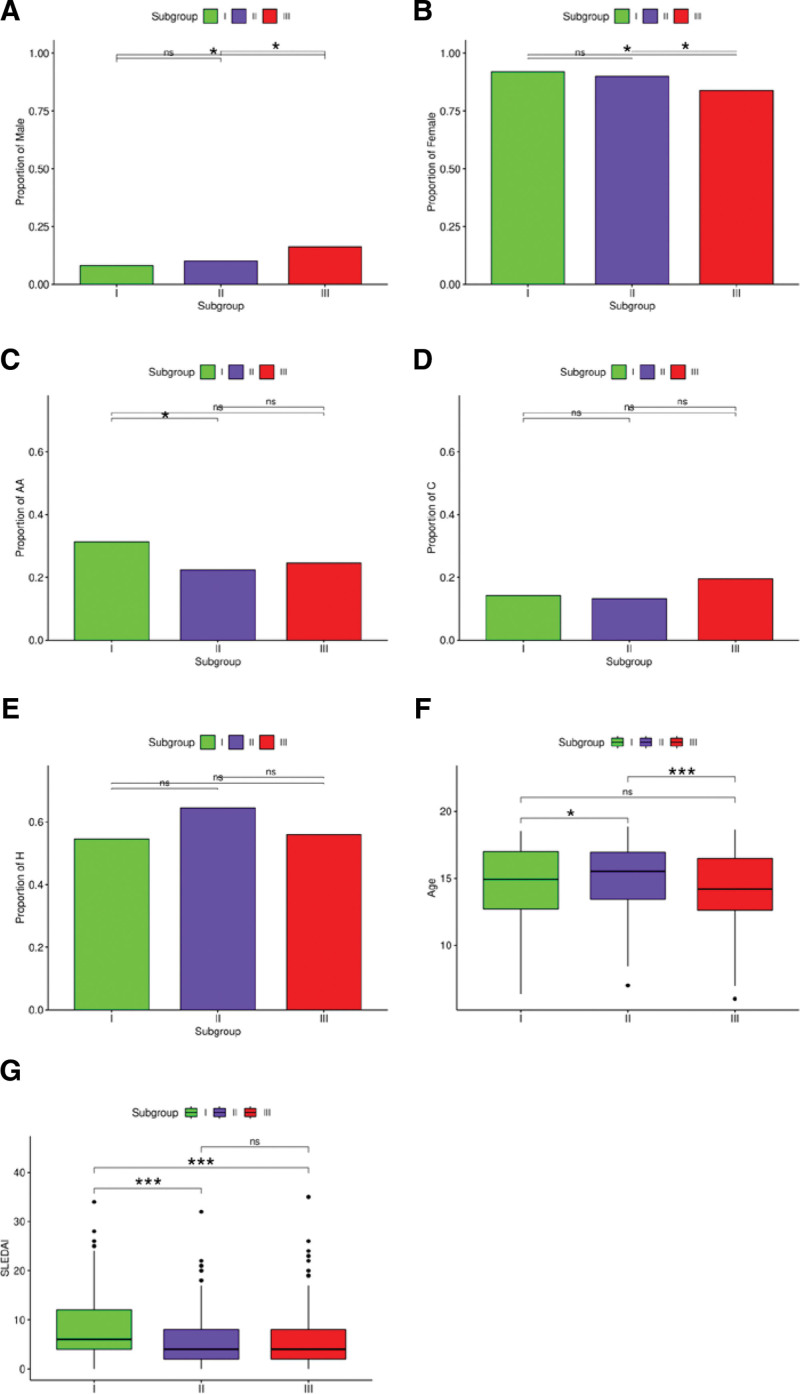
Analysis of the clinical characteristics of subgroups. (A–E) Graphs showing the proportion of males, females, African Americans (AAs), Caucasians (Cs), and Hispanics (Hs) in each subgroup, respectively. The different colors on the abscissa represent different subgroups, and the ordinate represents the proportion. Differences in (F) age and differential analysis of (G) Systemic lupus erythematosus disease activity index (SLEDAI) in each subgroup. The results are shown in box plots. *P < .05, **P < .01, ***P < .001. ns = not significant.

In total, the three subgroups comprised 902 female and 129 male patients. The numbers of patients belonging to the three races were as follows: AA, 259; C, 158; and H, 576. After determining the proportions of patients of different sexes and races in the subgroups, we observed significant differences in the proportion of male and female patients between subgroups I and III, and between subgroups II and III (Fig. 2A and B). Moreover, the proportion of AAs differed significantly between subgroups I and II, whereas there was no significant difference between the proportion of Cs and Hs between subgroups (Fig. 2C–E).

To assess relationships between age and SLEDAI in the subgroups, we calculated the mean ± SD for patient age; the values obtained were as follows: subgroup I, 15.24 ± 5.10 years; subgroup II, 16.09 ± 5.76 years; and subgroup III, 15.09 ± 6.00 years. We then analyzed enrichment in terms of age in each subgroup, with significant differences observed between subgroups I and II, and between subgroups II and III (Fig. 2F). Similarly, the mean ± SD of SLEDAI was as follows: subgroup I, 4.03 ± 3.11; subgroup II, 1.88 ± 2.80; and subgroup III, 2.68 ± 3.21. These findings showed that the SLEDAI of subgroup I differed significantly from that of subgroups II and III (*P* < .05) (Fig. 2G).

### 3.4. DEGs in each subgroup and PPI network analysis

**Figure 3.**
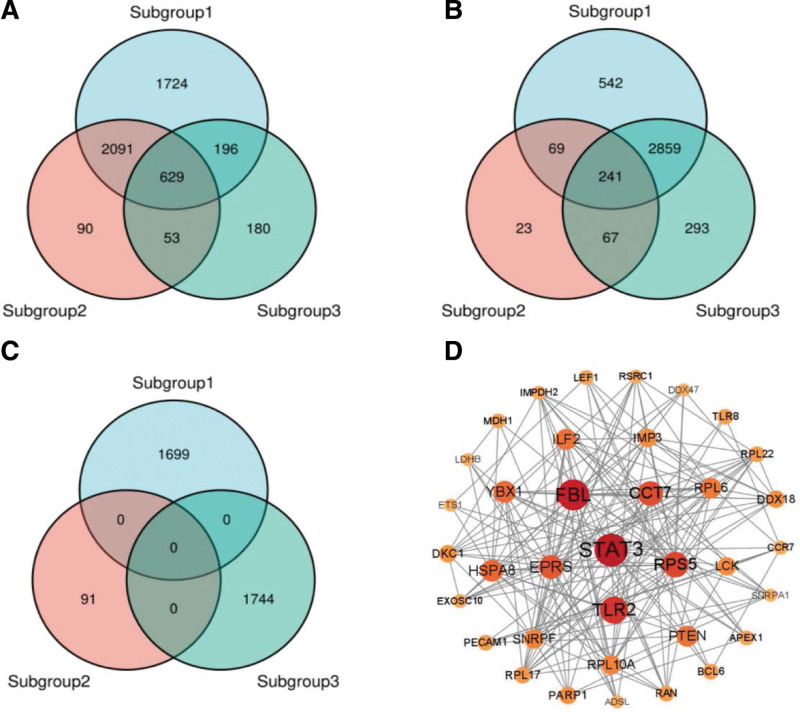
Differentially expressed genes (DEGs) for each subgroup. (A) DEGs were screened between subgroups and normal controls, and (B) subgroup DEGs were compared. (C) Subgroup-specific DEGs. The different colors represent different subgroups, and the numbers in the circles represent the number of genes. (D) Protein–protein interaction (PPI) network of genes with >10 hubs. A darker color and larger shape of the circle signify a larger number of nodes.

We then compared the three subgroups with the control group to screen for DEGs (Fig. 3A) and identified overlapping DEGs between the subgroups (Fig. 3B). To distinguish specific genes in each subgroup, we screened DEGs that demonstrated upregulated expression in specific subgroups (Fig. 3C) and confirmed the absence of overlap among these DEGs between subgroups. This process identified 3534 specific upregulated genes: subgroup I, 1699; subgroup II, 91; and subgroup III, 1744. Table 1 lists the top 10 specifically upregulated genes in each subgroup. We then screened the genes associated with more hubs in the three subgroups (Fig. 3D), resulting in the identification of 10 genes with the largest number of nodes (*STAT3, FBL, TLR2, RPS5, CCT7, EPRS, HSPA8, YBX1, ZLF2,* and *PTEN*), representing the genes possibly expressed to perform prominent functions (Table 2).

**Table 1:** List of the top 10 specifically upregulated genes in each subgroup.

Subgroup I	Subgroup II	Subgroup III
*REPS2*	*RIOK3*	*OCIAD2*
*MTMR3*	*AP2M1*	*IMP3*
*IL18R1*	*TSPAN5*	*SAE1*
*KIAA0319L*	*YBX1*	*SNRPF*
*NEDD9*	*RXRA*	*ZNF22*
*KCNJ15*	*PDZK1IP1*	*DDX18*
*MANSC1*	*FBXO7*	*LEF1*
*PDLIM7*	*GSPT1*	*PARP1*
*TGFA*	*MAF1*	*MIF*
*SLC26A8*	*RAB2B*	*LCK*

**Table 2:** Top 10 DEGs with the most hubs.

Gene	Degree	Subgroup
*STAT3*	32	I
*FBL*	30	III
*TLR2*	26	I
*RPS5*	24	III
*CCT7*	23	III
*EPRS*	22	III
*HSPA8*	20	III
*YBX1*	20	II
*ILF2*	19	III
*PTEN*	19	I

DEG = differentially expressed gene.

### 3.5. GSEA

**Figure 4.**
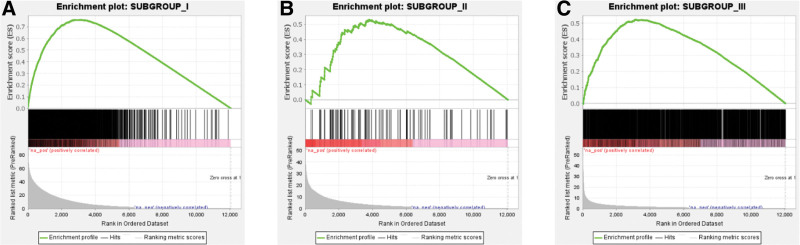
Gene set enrichment analysis (GSEA) of specific differentially expressed genes (DEGs) in each subgroup versus normal controls. (A–C) The three subgroups. The green line represents the gene-enrichment score, each black line represents a gene, and the gray area represents the signal-to-noise ratio between each respective subgroup and the control group.

We performed GSEA to analyze unique DEGs between different subgroups and determined the consistency of DEGs between subgroups and normal samples (Fig. 4A–C). Results showing *P* values and false discovery rates of < .01 suggested consistency in the uniqueness of the DEGs between different subgroups and that of the DEGs between subgroups and normal samples.

### 3.6. WGCNA results

**Figure 5.**
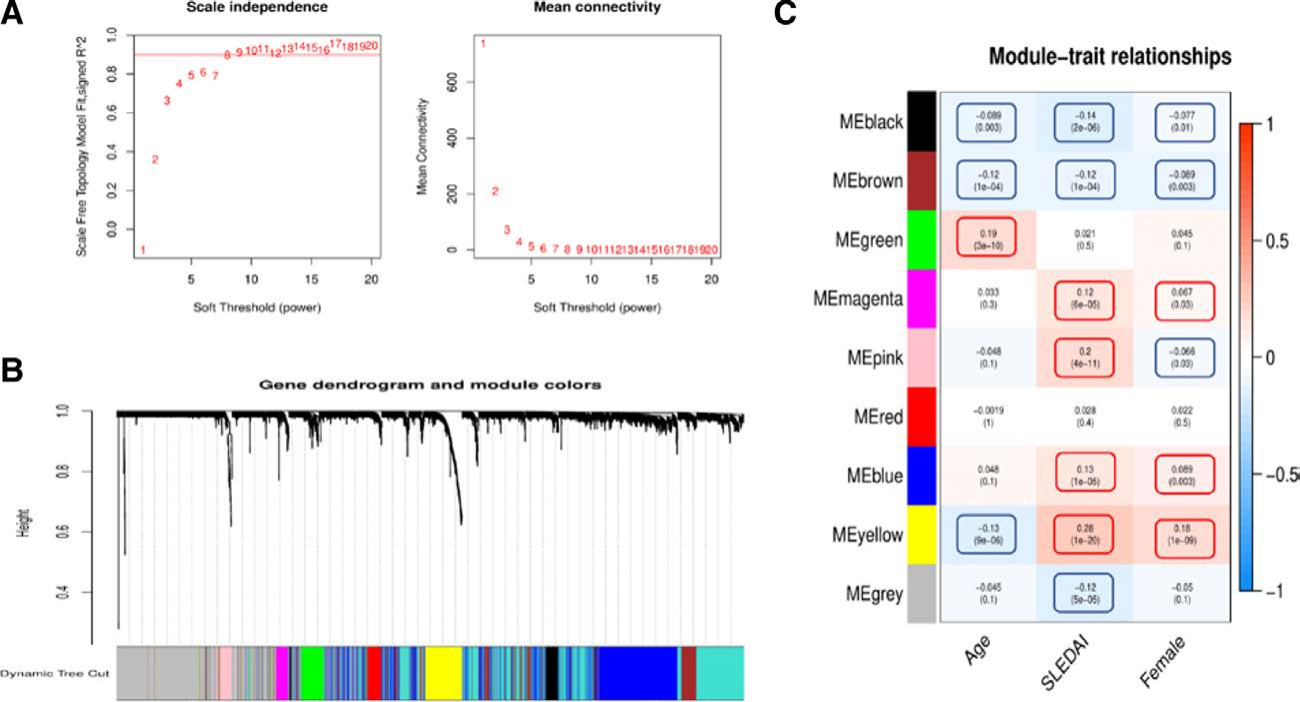
Weighted gene co-expression network analysis. (A) The graph on the left shows the relationship between the power value and the scale-free fitting index, and the graph on the right shows the relationship between the power value and the average connectivity. The red line represents the optimal threshold. (B) The dynamic tree method was used to form the original color modules of gene clusters. The upper part is the branch of the tree representing the gene, and the lower part is the gene corresponding to the color module. (C) Heat map of the correlation between clinical features and color modules. The ordinate represents different color modules and the abscissa represents the clinical features. The red color represents high expression of clinical features within the color modules and the blue color represents low expression. The numbers in parentheses represent the *P* values. SLEDAI = systemic lupus erythematosus disease activity index.

WGCNA performed on 12,062 genes between subgroups revealed a gene connectivity resembling a scale-free network at a power value of <8.0 (Fig. 5A). After screening, nine gene color modules were obtained using the dynamic tree method (Fig. 5B): black, 89; brown, 1252; green, 132; magenta, 71; pink, 86; red, 96; blue, 753; yellow, 225; and gray, 830. We selected the top three genes with the largest differences in each color module (Table 3) to analyze the relationship between the modules and clinical traits, followed by the analysis of gene color modules in terms of age, SLEDAI, and sex to obtain correlations among these parameters in each gene module (Fig. 5C). We observed that age was positively correlated with the green gene module, and negatively correlated with the black, brown, and yellow modules. SLEDAI was positively correlated with the magenta, pink, blue, and yellow modules, and negatively correlated with the black, brown, and gray modules. Females were positively correlated with the magenta, blue, and yellow modules, whereas males were positively correlated with the black, brown, and pink modules.

**Table 3:** Top three genes showing the most significant differential expression in each color module.

Black	Brown	Green	Magenta	Pink	Red	Blue	Yellow	Gray
*AASDHPPT*	*AARS*	*ABCC13*	*ABCC3*	*ABCA13*	*ANP32A*	*ABCA7*	*ABCA1*	*AAK1*
*AKAP11*	*ABCB7*	*ABCC4*	*ABLIM3*	*ACACB*	*APOBEC3A*	*ABCC5*	*ACOT9*	*AASDH*
*ALG5*	*ABCE1*	*ADORA1*	*ACRBP*	*ACP6*	*ARPC5*	*ABHD2*	*ACSL3*	*ABCB1*

**Figure 6.**
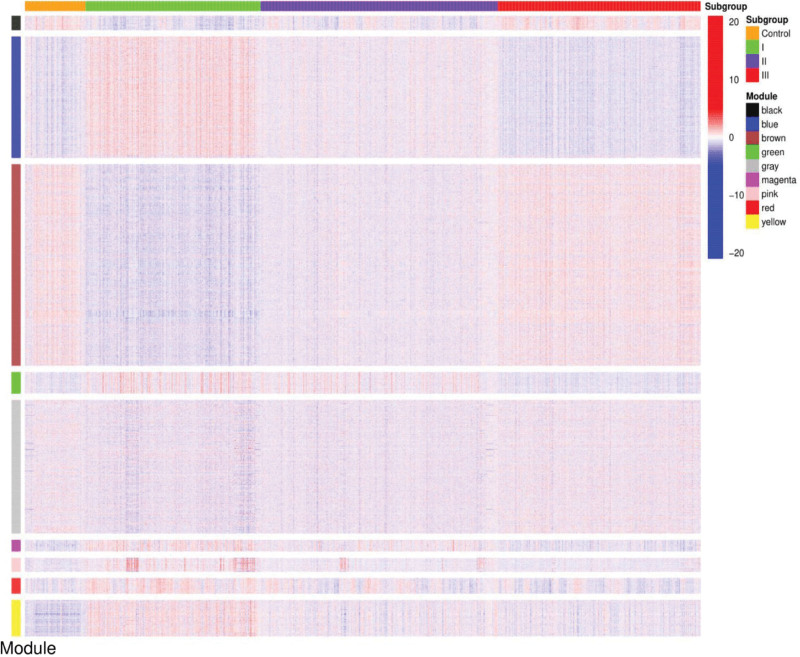
Heat map showing the correlation of subgroups with the color modules. The ordinate represents nine different color modules, and the abscissa represents the normal control group and the three subgroups. The blue color represents the subgroup with low expression within the color module and the red color represents the subgroup with high expression.

The gene color module can be used to correlate clinical features with gene subgroups. After intersecting the gene color module with the genotyping results, we obtained a heat map of the correlations between gene modules and gene subgroups (Fig. 6). Subgroup I genes were poorly expressed in the black, brown, and gray modules, and highly expressed in the other modules, which contrasted with that observed in the normal control group. Subgroup II genes were highly expressed in the green module, and there were no significant differences in the other color modules. Finally, the expression of subgroup III genes in each color module was identical to that observed in the normal control group.

### 3.7. GO and KEGG analyses

**Figure 7.**
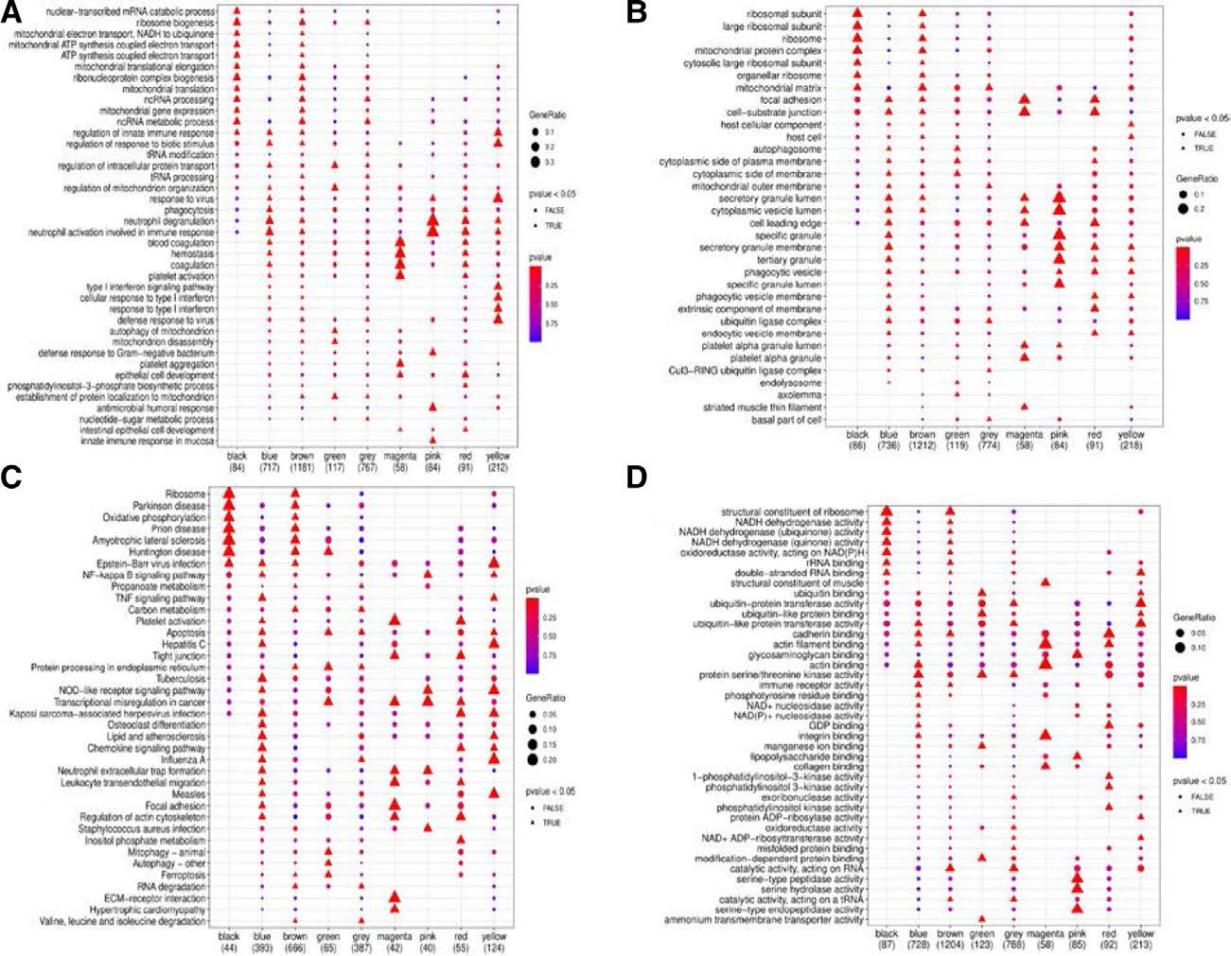
Results of gene ontology (GO) and Kyoto Encyclopedia of Genes and Genomes (KEGG) enrichment analyses of the gene color modules. (A–D) Bubble diagrams of the relationship between biological process (BP), cellular component (CC), molecular function (MF), and KEGG pathways with the gene modules. The abscissa represents various colored gene modules, and the ordinate represents various biological aspects. The circle represents a *P* > .05, and the triangle represents a *P* < .05. A larger shape and darker color represent a higher enrichment ratio and smaller *P* value. ECM = extracellular matrix.

GO can be used to analyze the association of each gene module with BP, CC, and MF, and indirectly determine the relationship between subgroups and biological characteristics. Blood coagulation, hemostasis, coagulation, platelet activation, and platelet aggregation were the BPs that were found to be closely related to the magenta module (Fig. 7A). The secretory granule lumen, cytoplasmic vesicle lumen, special granules, secretory granule membranes, tertiary granules, and special granule lumen were the CCs found to be associated with the pink modules (Fig. 7B). The magenta modules were associated with the following MFs: structural constituents of muscle, actin filament binding, actin binding, integrin binding, and collagen binding (Fig. 7C). Excluding the enrichment of KEGG pathways in the same color module, the most significantly enriched pathways in each color module were as follows: ribosome, tuberculosis, prion disease, autophagy, valine, leucine, and isoleucine degradation, extracellular matrix-receptor interaction, transcriptional dysregulation in cancer, platelet activation, and influenza A (Fig. 7D).

**Figure 8.**
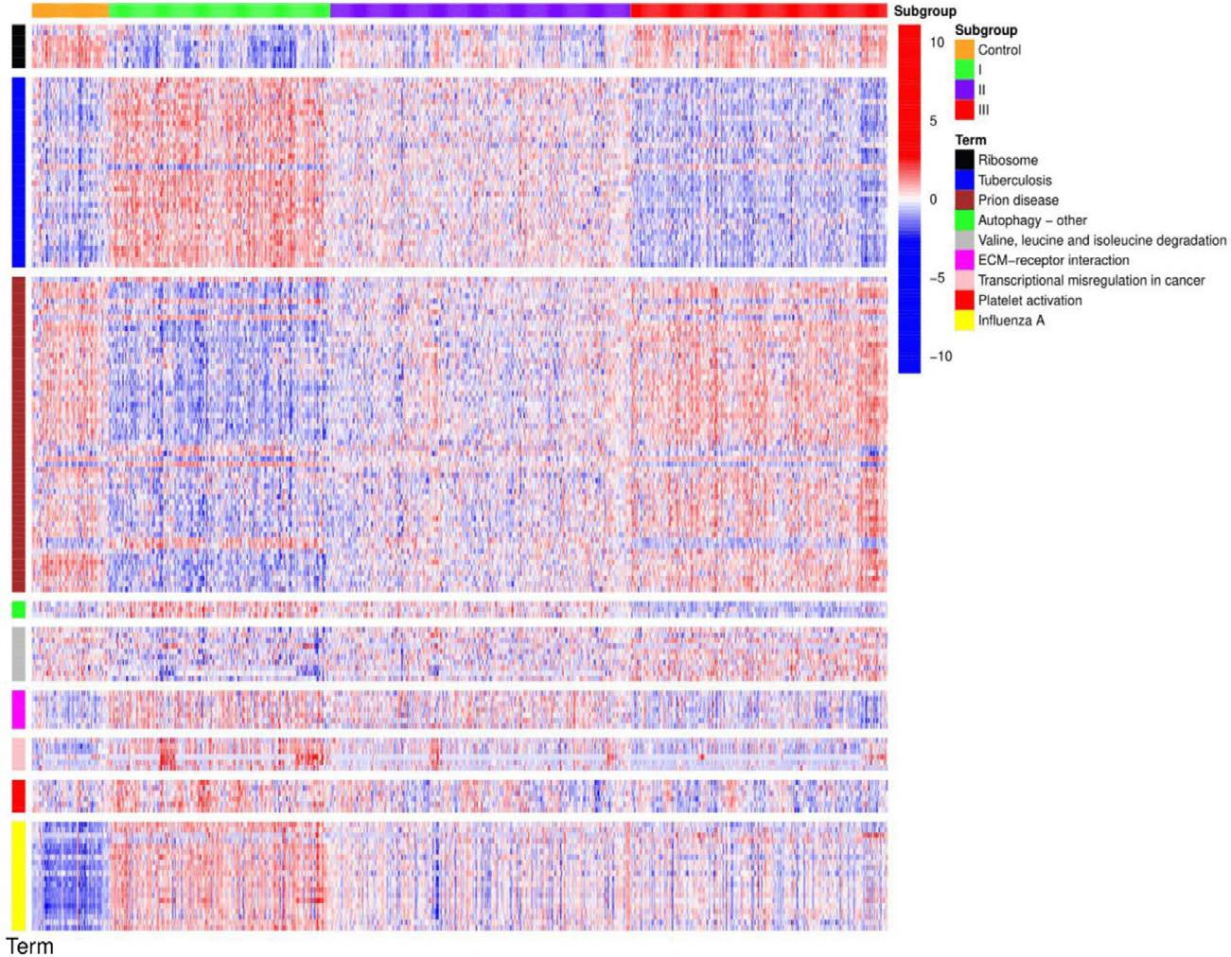
Heat map of subpopulation and gene color module correlations. The ordinates represent representative pathways in gene modules with different colors, and the abscissas represent different subgroups. Blue color represents pathways with low expression in subgroups, and red color represents pathways with high expression. ECM = extracellular matrix.

We then analyzed the nine pathways obtained from each module by comparing the normal control group with the three subgroups and generating a heat map of the results (Fig. 8). In the present study, only the most prominently expressed pathways were selected. High expression of ribosome, prion disease, and valine, leucine, and isoleucine degradation, and low expression of tuberculosis, autophagy–other, extracellular matrix–receptor interaction, transcriptional dysregulation in cancer, platelet activation, and influenza A were observed in the normal controls and subgroup III. Contrary to the results for subgroup I, no significant difference was observed in the expression of any pathway in subgroup II. These results corroborate our WGCNA results.

### 3.8. Summary of the characteristics of each subgroup

We then evaluated the clinical characteristics, specifically upregulated genes, and the results of GO and KEGG analyses for each subgroup (Table 4). Clinical features showed that high levels of SLEDAI and female patients were mainly concentrated in subgroup I, whereas male patients and low levels of SLEDAI were concentrated in subgroup II. We then aggregated the listed terms for the BP, CC, and MF pathways and KEGG analyses that were significantly expressed in each subgroup.

**Table 4:** Summary of subgroups.

Category	Significantly enriched terms
Subgroup I	
Clinical features	SLEDAI (High)
	Female
Specific gene	*REPS2, MTMR3, IL18R1, STAT3, TLR2, PTEN*
BP	Neutrophil degranulation, Regulation of mitochondrion organization, Blood coagulation, Defense response to virus
CC	Specific granule, Autophagosome, Platelet alpha granule, Focal adhesion, Phagocytic vesicle membrane
MF	Protein serine/threonine kinase activity, Ammonium transmembrane transporter activity, Actin filament binding, Serine-type endopeptidase activity, 1-phosphatidylinositol-3-kinase activity, Double-stranded RNA binding
KEGG	Tuberculosis, Autophagy − other, ECM − receptor interaction, Transcriptional misregulation in cancer, Platelet activation, Influenza A
Subgroup II	
Clinical features	SLEDAI (Low)
Specific gene	*RIOK3, AP2M1, TSPAN5, YBX1, RXRA*
BP	Regulation of mitochondrion organization
CC	Autophagosome
MF	Ammonium transmembrane transporter activity
Subgroup III	
Clinical features	Male
Specific gene	*OCIAD2, IMP3, SAE1, SNRPF, ZNF22, MIF, FBL, RPS5, CCT7, HSPA8, ILF2*
BP	Nuclear-transcribed mRNA catabolic process, Nonsense-mediated decay, Ribonucleoprotein complex biogenesis, ncRNA processing
CC	Ribosomal subunit, Mitochondrial matrix
MF	Structural constituent of ribosome, catalytic activity, acting on RNA
KEGG	Ribosome; Prion disease; Valine, leucine, and isoleucine degradation

BP = biological process, CC = cellular component, ECM = extracellular matrix, KEGG = Kyoto Encyclopedia of Genes and Genomes, MF = molecular function, SLEDAI = systemic lupus erythematosus disease activity index.

## 4. Discussion

SLE is caused by a combination of environmental and genetic risk factors.^[7]^ To obtain insights regarding cSLE genotyping, we used an unsupervised learning algorithm and analyzed the transcriptome data of patients with cSLE. We obtained three subgroups of gene clusters that were compared from different perspectives, including clinical characteristics and specific DEGs. GO and KEGG analyses were then performed to understand the characteristics of the subgroups at the biological level, which provided the basis for understanding the cSLE subgroups.

Our study included considerably more women than men, which is consistent with the higher prevalence of SLE in women than in men. Banchereau et al^[14]^ observed racial differences in SLE severity, with AAs often showing a more severe disease than Cs. We observed that AA patients were mainly concentrated in subgroup I. SLEDAI is a common indicator used to clinically evaluate SLE condition and treatment, with a larger SLEDAI value representing more serious conditions.^[15]^ Figure 2G shows that SLEDAI was the highest in subgroup I and lowest in subgroup II, indicating SLEDAI as a helpful indicator for distinguishing different subgroups. This observation, combined with the results of ethnic enrichment, indicates that patients in subgroup I may have a more severe disease. Figure 5C shows that the proportion of females agreed with the SLEDAI value in subgroup I. The proportion of women in subgroup I was high, and male patients were mainly enriched in the black and brown gene modules, corresponding to subgroup III.

Interleukin-18 (IL-18) is a proinflammatory cytokine belonging to the IL-1 family. Proinflammatory cytokine signaling by nuclear factor-κB and mitogen-activated kinase induces interferon (IFN)-γ production in autoimmune diseases, resulting in their designation as IFN-γ-inducing factors.^[16]^ IL-18 levels are significantly elevated in active SLE and LN,^[17,18]^ and a previous study suggested that IL-18 and IL-12 promote T helper (Th)1 cell activation, thereby increasing the production of the Th1 cytokine IFN-γ, inducing nitric oxide synthesis, and mediating nitric oxide production in glomerulonephritis and vasculitis.^[18]^ Additionally, Wu et al^[19]^ showed that IL-18 is the most unfavorable factor leading to poor clinical outcomes in long-term renal therapy in patients with cSLE, suggesting that IL-18 may serve as a potential target for the treatment of LN.

Phosphoinositide 3-kinase (PI3K) plays an important role in the development of LN.^[20,21]^ Wang et al^[22]^ reported that the injection of the selective PI3K p110δ (PI3Kδ) inhibitor IC87114 into a mouse model of lupus improved renal function and survival. Another study in a mouse model of systemic lupus showed that PI3Kδ reduced kidney infiltration of macrophages and ameliorated lupus-like symptoms.^[23]^ These findings indicated that PI3K inhibitors might be effective for treating LN patients.

The gene encoding myotubularin-related protein 3 (*MTMR3*) is an autophagy-related gene that was downregulated in a genome-wide analysis of kidney biopsy samples from LN patients.^[24]^ In the present study, we found that *MTMR3* was highly expressed in subgroup I. Although subgroup I was not associated with LN, members of this subgroup were associated with the autophagy pathway.

Signal transducer and activator of transcription 3 (STAT3) plays a key role in regulating inflammation and the immune response.^[25]^ STAT3 is activated by phosphorylation of tyrosine 705 and/or serine 727, and phosphorylated STAT3 can bind target sequences in promoter regions to regulate gene transcription.^[26]^ Recently, elevated STAT3 levels in SLE have attracted increased attention. Arakawa et al^[27]^ detected a significantly increased expression of STAT3 and phosphorylated STAT3 in the kidneys of patients with LN. In the present study, *STAT3* was the most nodal gene in the PPI network and was specifically upregulated in subgroup I. Based on these findings, we speculated that subgroup I might be closely related to LN. Moreover, a recent study reported that STAT3 phosphorylation can mediate the effect of IFN-α on B-cell differentiation and activation in SLE,^[28]^ which may lead to the identification of new targets for SLE treatment.

Y-box binding protein 1 (YB-1/YBX1) is a transcription factor that regulates the expression of multiple genes by interacting directly or indirectly with the Y-box sequence in gene promoters.^[29]^ Elevated YB-1 expression is related to SLE vasculitis; however, the specific mechanisms underlying these phenomena remain unclear.^[30]^

The ribosomal pathway is closely related to SLE.^[31,32]^ A previous study showed that 15% to 35% of patients with SLE harbor ribosomal P antibodies, which are highly specific and detected in most cases of cSLE.^[33]^ These antibodies promote the production of proinflammatory cytokines, leading to central nervous system damage, and are closely related to SLE neuropsychiatric manifestations, especially psychosis.^[34]^

Macrophage migration-inhibitory factor (MIF) is an immunomodulatory mediator that is widely expressed in tissues and is associated with the development of neurological diseases.^[35]^ Li et al^[36]^ reported that MIF affected tau hyperphosphorylation in a mouse model by promoting the expression of other inflammatory mediators, including astrocyte activation and subsequent release of soluble cytokines, resulting in nerve cell damage. In the present study, we found that subgroup III included ribosome-related pathways, suggesting a possible association between the genes in this subgroup with neuropsychiatric symptoms in lupus.

Heat-shock protein family A member 8 (HSPA8) is a molecular chaperone involved in various cellular processes and plays a critical regulatory role in chaperone-mediated autophagy and immune-related diseases.^[37]^ Autophagy, a process by which lysosomes degrade intracellular components, plays an important role in the central nervous system by promoting neuronal homeostasis.^[38]^ Neurological dysfunction damages Brodmann area 22 in the brain, which is associated with autophagy gene expression, resulting in reduced mRNA levels of some autophagy-related genes.^[39]^ By analyzing HSPA8-associated polymorphisms in patients with schizophrenia and healthy individuals, Bozidis et al^[40]^ found that HSPA8 polymorphisms and neuroticism levels were the variables most strongly and independently associated with psychiatric disorders compared with those in healthy controls. In the present study, we identified HSPA8 in subgroup III, further supporting a possible link between members of this subgroup and lupus neuropsychiatric symptoms.

Here, we investigated the possible types and therapeutic targets of cSLE at the molecular level. This study has certain limitations. Recent studies have shown that the transcriptomic classification of cSLE remains incomplete. Thus, the relationships between SLE subgroups and cSLE pathogenesis and pathophysiological processes must be verified experimentally.^[41]^ Second, efforts to classify cSLE require the combined results of proteomics and metabolomics analyses. Additionally, more evidence is required to analyze the relationships between DEG subgroups and clinical characteristics. Therefore, the precise treatment of cSLE remains challenging.

## 5. Conclusion

In summary, transcriptome data were used to divide patients with cSLE into three subgroups, followed by the evaluation of the clinical characteristics and DEGs in each subgroup. We found that IL-18, PI3K, MTMR3, and STAT3 are potential therapeutic targets for subgroup I, and MIF and HSPA8 are potential therapeutic targets for subgroup III. These observations provide molecular evidence to support the development of diagnostic methods and individualized treatments for cSLE.

## Acknowledgments

This work was supported by the Medical Discipline Leader of the Yunnan Provincial Commission of Health and Family Planning (no: D-2017057), Yunnan Provincial Key Laboratory of Reproductive Health Research of Department of Education, Graduate Tutor Team of Obstetrics and Gynecology of Yunnan Provincial Department of Education, and The Key Construction Disciplines of The First Affiliated Hospital of Dali University.

## Author contributions

**Conceptualization:** Yuanyuan Zhang, Guangming Wang.

**Data curation:** Weijiang Chu, Pengyu Wang.

**Formal analysis:** Huaqiu Chen.

**Funding acquisition:** Guangming Wang.

**Methodology:** Jianglei Ma, Huijie Zhang.

**Software:** Jianglei Ma, Huijie Zhang.

**Writing – original draft:** Jianglei Ma, Huijie Zhang.

**Writing – review & editing:** Yuanyuan Zhang, Guangming Wang.

## References

[1] Smith EMD Lythgoe H Midgley A. Juvenile-onset systemic lupus erythematosus: update on clinical presentation, pathophysiology and treatment options. Clin Immunol. 2019;209:108274. 31678365 10.1016/j.clim.2019.108274

[2] Harry O Yasin S Brunner H. Childhood-onset systemic lupus erythematosus: a review and update. J Pediatr. 2018;196:22–30.e2. 29703361 10.1016/j.jpeds.2018.01.045

[3] Torell F Eketjäll S Idborg H. Cytokine profiles in autoantibody defined subgroups of systemic lupus erythematosus. J Proteome Res. 2019;18:1208–17. 30742448 10.1021/acs.jproteome.8b00811

[4] Subspecialty Group of Immunology, the Society of Pediatrics, Chinese Medical Association, Authorial Board, Chinese Journal of Pediatrics. Chinese guidelines for the diagnosis and treatment of childhood-onset systemic lupus erythematosus. Chin J Pediatr. 2021;59:1009–24. 10.3760/cma.j.cn112140-20210905-00743 34856659

[5] Larosa M Iaccarino L Gatto M. Advances in the diagnosis and classification of systemic lupus erythematosus. Expert Rev Clin Immunol. 2016;12:1309–20. 27362864 10.1080/1744666X.2016.1206470

[6] Kwon YC Chun S Kim K. Update on the genetics of systemic lupus erythematosus: genome-wide association studies and beyond. Cells. 2019;8:1180. 31575058 10.3390/cells8101180 PMC6829439

[7] Webber D Cao J Dominguez D. Association of systemic lupus erythematosus (SLE) genetic susceptibility loci with lupus nephritis in childhood-onset and adult-onset SLE. Rheumatology (Oxford). 2020;59:90–8. 31236574 10.1093/rheumatology/kez220

[8] Nehar-Belaid D Hong S Marches R. Mapping systemic lupus erythematosus heterogeneity at the single-cell level. Nat Immunol. 2020;21:1094–106. 32747814 10.1038/s41590-020-0743-0 PMC7442743

[9] Grech P Khamashta M. Targeted therapies in systemic lupus erythematosus. Lupus. 2013;22:978–86. 23963429 10.1177/0961203313499417

[10] Zhang B Zeng K Li R. Construction of the gene expression subgroups of patients with coronary artery disease through bioinformatics approach. Math Biosci Eng. 2021;18:8622–40. 34814316 10.3934/mbe.2021427

[11] Wilkerson MD Hayes DN. ConsensusClusterPlus: a class discovery tool with confidence assessments and item tracking. Bioinformatics. 2010;26:1572–3. 20427518 10.1093/bioinformatics/btq170 PMC2881355

[12] Su G Morris JH Demchak B. Biological network exploration with Cytoscape 3. Curr Protoc Bioinformatics. 2014;47:8.13.1–8.13.24. 10.1002/0471250953.bi0813s47 PMC4174321 25199793

[13] Langfelder P Horvath S. WGCNA: an R package for weighted correlation network analysis. BMC Bioinform. 2008;9:559. 10.1186/1471-2105-9-559 PMC2631488 19114008

[14] Banchereau R Hong S Cantarel B. Personalized immunomonitoring uncovers molecular networks that stratify lupus patients. Cell. 2016;165:551–65. 27040498 10.1016/j.cell.2016.03.008 PMC5426482

[15] Gladman DD Ibañez D Urowitz MB. Systemic lupus erythematosus disease activity index 2000. J Rheumatol. 2002;29:288–91. 11838846

[16] Aringer M. Inflammatory markers in systemic lupus erythematosus. J Autoimmun. 2020;110:102374. 31812331 10.1016/j.jaut.2019.102374

[17] Hu D Liu X Chen S. Expressions of IL-18 and its binding protein in peripheral blood leukocytes and kidney tissues of lupus nephritis patients. Clin Rheumatol. 2010;29:717–21. 20140691 10.1007/s10067-010-1386-6

[18] Wong CK Ho CY Li EK. Elevated production of interleukin-18 is associated with renal disease in patients with systemic lupus erythematosus. Clin Exp Immunol. 2002;130:345–51. 12390326 10.1046/j.1365-2249.2002.01989.x PMC1906516

[19] Wu CY Yang HY Yao TC. Serum IL-18 as biomarker in predicting long-term renal outcome among pediatric-onset systemic lupus erythematosus patients. Medicine (Baltim). 2016;95:e5037. 10.1097/MD.0000000000005037 PMC5059068 27749566

[20] Puri KD Gold MR. Selective inhibitors of phosphoinositide 3-kinase delta: modulators of B-cell function with potential for treating autoimmune inflammatory diseases and B-cell malignancies. Front Immunol. 2012;3:256. 22936933 10.3389/fimmu.2012.00256 PMC3425960

[21] Yanaba K Bouaziz JD Matsushita T. B-lymphocyte contributions to human autoimmune disease. Immunol Rev. 2008;223:284–99. 18613843 10.1111/j.1600-065X.2008.00646.x

[22] Wang Y Zhang L Wei P. Inhibition of PI3Kdelta improves systemic lupus in mice. Inflammation. 2014;37:978–83. 24445960 10.1007/s10753-014-9818-0

[23] Suárez-Fueyo A Rojas JM Cariaga AE. Inhibition of PI3Kdelta reduces kidney infiltration by macrophages and ameliorates systemic lupus in the mouse. J Immunol. 2014;193:544–54. 24935930 10.4049/jimmunol.1400350

[24] Zhou XJ Nath SK Qi YY. Brief report: identification of MTMR3 as a novel susceptibility gene for lupus nephritis in northern Han Chinese by shared-gene analysis with IgA nephropathy. Arthritis Rheumatol. 2014;66:2842–8. 24943867 10.1002/art.38749 PMC4180767

[25] Hillmer EJ Zhang H Li HS. STAT3 signaling in immunity. Cytokine Growth Factor Rev. 2016;31:1–15. 27185365 10.1016/j.cytogfr.2016.05.001 PMC5050093

[26] Zhang Y Day K Absher DM. STAT3-mediated allelic imbalance of novel genetic variant Rs1047643 and B-cell-specific super-enhancer in association with systemic lupus erythematosus. Elife. 2022;11:e72837. 35188103 10.7554/eLife.72837 PMC8884724

[27] Arakawa T Masaki T Hirai T. Activation of signal transducer and activator of transcription 3 correlates with cell proliferation and renal injury in human glomerulonephritis. Nephrol Dial Transplant. 2008;23:3418–26. 18556750 10.1093/ndt/gfn314

[28] De Groof A Ducreux J Aleva F. STAT3 phosphorylation mediates the stimulatory effects of interferon alpha on B cell differentiation and activation in SLE. Rheumatology (Oxford). 2020;59:668–77. 31504941 10.1093/rheumatology/kez354

[29] Lyabin DN Eliseeva IA Ovchinnikov LP. YB-1 protein: functions and regulation. Wiley Interdiscip Rev RNA. 2014;5:95–110. 24217978 10.1002/wrna.1200

[30] Qin W Feng SG Deng HX. Serological screening of autoantigens associated with vasculitis in systemic lupus erythematosus [article in Chinese]. Sichuan Da Xue Xue Bao Yi Xue Ban. 2007;38:132–4, 149. 17294748

[31] Choi MY FitzPatrick RD Buhler K. A review and meta-analysis of anti-ribosomal P autoantibodies in systemic lupus erythematosus. Autoimmun Rev. 2020;19:102463. 31927088 10.1016/j.autrev.2020.102463

[32] Shi ZR Han YF Yin J. The diagnostic benefit of antibodies against ribosomal proteins in systemic lupus erythematosus. Adv Rheumatol. 2020;60:45. 32859277 10.1186/s42358-020-00148-2

[33] Viana VT Durcan L Bonfa E. Ribosomal P antibody: 30 years on the road. Lupus. 2017;26:453–62. 28394227 10.1177/0961203317690243

[34] Toubi E Shoenfeld Y. Clinical and biological aspects of anti-P-ribosomal protein autoantibodies. Autoimmun Rev. 2007;6:119–25. 17289545 10.1016/j.autrev.2006.07.004

[35] Chen X Chen Y Qi D. Multifaceted interconnections between macrophage migration inhibitory factor and psychiatric disorders. Prog Neuropsychopharmacol Biol Psychiatry. 2022;112:110422. 34358623 10.1016/j.pnpbp.2021.110422

[36] Li SQ Yu Y Han JZ. Deficiency of macrophage migration inhibitory factor attenuates tau hyperphosphorylation in mouse models of Alzheimer’s disease. J Neuroinflammation. 2015;12:177. 26382037 10.1186/s12974-015-0396-3 PMC4574615

[37] Bonam SR Ruff M Muller S. HSPA8/HSC70 in immune disorders: a molecular rheostat that adjusts chaperone-mediated autophagy substrates. Cells. 2019;8:849. 31394830 10.3390/cells8080849 PMC6721745

[38] Nixon RA. The role of autophagy in neurodegenerative disease. Nat Med. 2013;19:983–97. 23921753 10.1038/nm.3232

[39] Schneider JL Miller AM Woesner ME. Autophagy and schizophrenia: a closer look at how dysregulation of neuronal cell homeostasis influences the pathogenesis of schizophrenia. Einstein J Biol Med. 2016;31:34–9. 28239307 10.23861/EJBM201631752 PMC5321090

[40] Bozidis P Hyphantis T Mantas C. HSP70 polymorphisms in first psychotic episode drug-naive schizophrenic patients. Life Sci. 2014;100:133–7. 24548631 10.1016/j.lfs.2014.02.006

[41] Hubbard EL Grammer AC Lipsky PE. Transcriptomics data: pointing the way to subclassification and personalized medicine in systemic lupus erythematosus. Curr Opin Rheumatol. 2021;33:579–85. 34410228 10.1097/BOR.0000000000000833

